# A very old Blalock–Taussig shunt: a case report

**DOI:** 10.1093/ehjcr/ytae164

**Published:** 2024-03-28

**Authors:** Houssam Bendoudouch, Badre El Boussaadani, Zainab Raissuni, Samir Atmani

**Affiliations:** Cardiology Department, Mohamed VI University Hospital of Tangier, Street of Rabat, Km 17, BP 398, 90100 Tangier, Morocco; Abdelmalek Essaadi University Faculty of Medicine and Pharmacy, Street of Rabat, Km 17, 90100 Tangier, Morocco; Cardiology Department, Mohamed VI University Hospital of Tangier, Street of Rabat, Km 17, BP 398, 90100 Tangier, Morocco; Abdelmalek Essaadi University Faculty of Medicine and Pharmacy, Street of Rabat, Km 17, 90100 Tangier, Morocco; Cardiology Department, Mohamed VI University Hospital of Tangier, Street of Rabat, Km 17, BP 398, 90100 Tangier, Morocco; Abdelmalek Essaadi University Faculty of Medicine and Pharmacy, Street of Rabat, Km 17, 90100 Tangier, Morocco; Paediatric Cardiology Department, Hassan II University Hospital of Fez, Avenue Hassan II, BP 1835, Atlas, 30050 Fez, Morocco

We report the case of a 14-year-old lady with a history of pulmonary valve atresia, treated with Modified Blalock-Taussig shunt (MBTS) shunt at the age of two years upon diagnosis.^1^ The patient was included in a staged Fontan surgical programme, but the patient was lost to follow-up and did not benefit from the total palliative treatment. Twelve years later, the patient consulted for increased dyspnoea, fatigability, and cyanosis. The physical examination revealed marked weight loss, peripheral cyanosis (lips, fingers), as well as pulse oxygen saturation of 70%. Transthoracic echocardiogram showed very restrictive MBTS graft (*Figure 1A*) with high velocity. A thoracic CT angiography was inconclusive about the permeability of the shunt. Invasive angiography^2^ revealed an obstructed MBTS (*Figure 1C*). It also showed very large major aorto-pulmonary collateral arteries (MAPCA) (*Figure 1D*), supplying the pulmonary arteries network. Surgery for Fontan procedure as well as total closure of the MBTS was indicated.^3^ On follow-up consultation following surgery, the patient displayed decreased dyspnoea, no marked cyanosis, and oxygen pulsed saturation of 85%.

**Figure 1:**
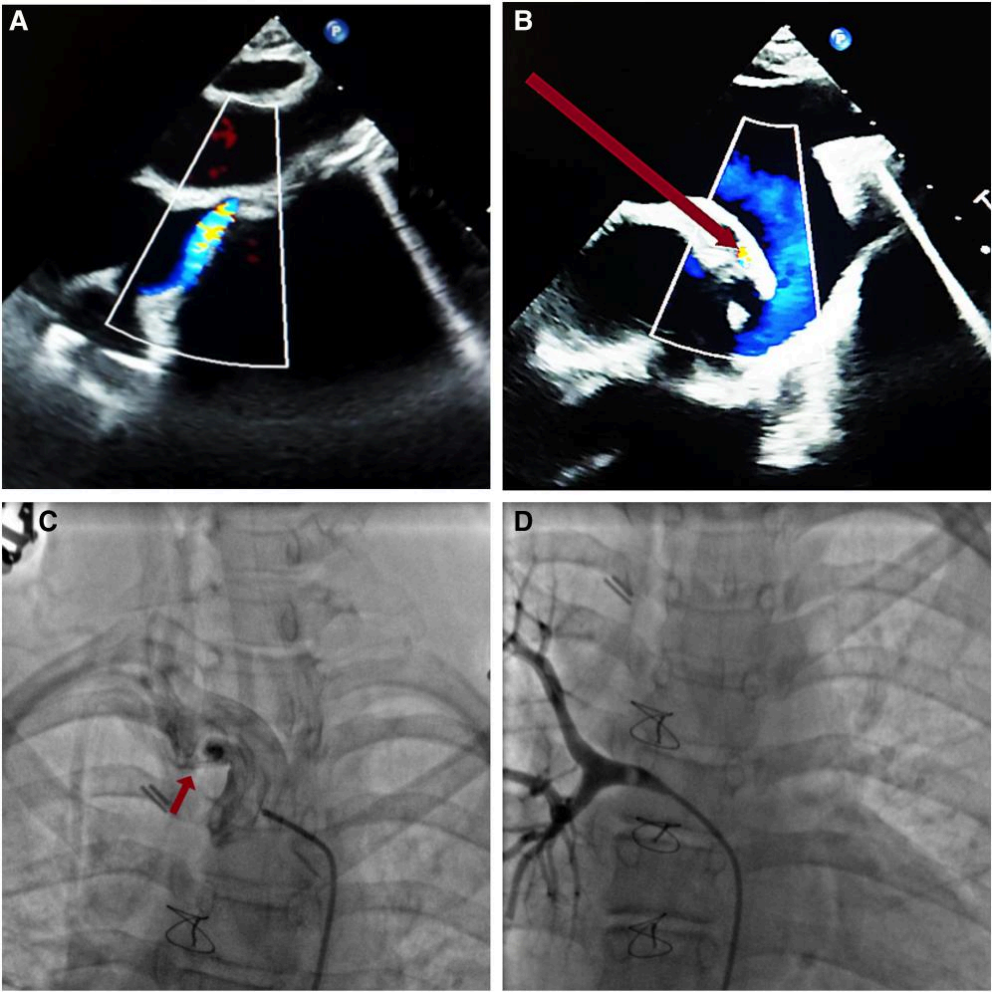
(*A* and *B*) Suprasternal view in transthoracic echocardiography showing aliasing in the MBTS (*A*) and major aorto-pulmonary collateral artery (*B*, arrow). (*C and D*) Thoracic angiography showing obstructed MBTS (*C*) and right MAPCA (*D*, arrow).

## Data Availability

The data underlying this article will be shared on reasonable request to the corresponding author.

## Lead author biography

Houssam Bendoudouch is a resident in the Cardiology Department at the Mohamed VI University Hospital of Tangier.

## Notes

**Consent:** The patient signed written informed consent for all procedures. The journal consent form has been signed by the patient. Patient information is provided anonymously in accordance with the Committee on Publication Ethics (COPE) guidelines.

**Funding**: None declared.
